# Supplementary material for: Identification of photoperiod-induced specific miRNAs in the adrenal glands of Sunite sheep (Ovis aries)
Source: Front Vet Sci. 2022 Jul 22;9:888207. doi: 10.3389/fvets.2022.888207 (PMC9354845; doi:10.3389/fvets.2022.888207)
Supplement: Supplementary file 1 [file Data_Sheet_1.ZIP › Supplementary file/miRDeep2 file.zip!/miRDeep2 file/Know_Structure/SP42A1/oar-mir-665.pdf]

miRBase precursor : oar-mir-665
Total read count : 35
oar-miR-665-5p read count : 0
oar-miR-665-3p read count : 30
remaining reads : 5

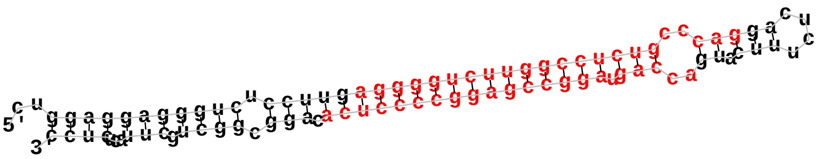

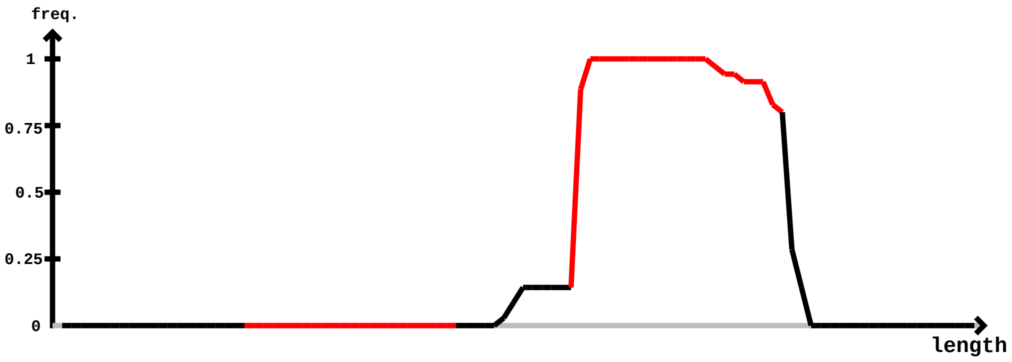

oar-miR-665-3p
oar-miR-665-5p

```
5'- cuggaggagggucuccuugaggggucuuggccucugcccaggacucuuucaugaccaguaggccgaggccccucacaggcggcugcuuacucucc   -3'   exp
    ..((((((((((.((((((((((((((((((((((..(((((....))).))..))).)))))))))))))))))).))).)))).))).))))..    reads   mm   sample
    .............................................cuuucaugaccaguaggccgagg...........................    1       0    seq
    ..............................................uuucaugaccaguaggccgaggccccu......................    2       0    seq
    ...............................................uucaugaccaguaggccgag............................    1       0    seq
    ...............................................uucaugaccaguaggccgaggcc.........................    1       0    seq
    .....................................................accaguaggccgaggccccu......................    1       0    seq
    .....................................................accaguaggccgaggccccuc.....................    1       0    seq
    .....................................................accaguaggccgaggccccuca....................    17      0    seq
    .....................................................accaguaggccgaggccccucac...................    4       0    seq
    .....................................................accaguaggccgaggccccucaca..................    3       0    seq
    ......................................................ccaguaggccgaggccccuca....................    1       0    seq
    ......................................................ccaguaggccgaggccccucac...................    1       0    seq
    ......................................................ccaguaggccgaggccccucaca..................    2       0    seq
```
